# Supplementary material for: Discovery and pharmacophoric characterization of chemokine network inhibitors using phage-display, saturation mutagenesis and computational modelling
Source: Nat Commun. 2023 Sep 16;14:5763. doi: 10.1038/s41467-023-41488-z (PMC10505172; doi:10.1038/s41467-023-41488-z)
Supplement: Supplementary file 4 — Source Data [file 41467_2023_41488_MOESM4_ESM.zip › SourceData/scripts/xoctet_20230808081637.tar!/xoctet/analysis/kinetic/SV_E32_sHD2_CCL8_kinetic_plot.pdf]

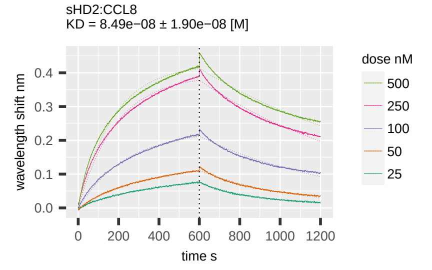
sHD2:CCL8
KD = 8.49e−08 ± 1.90e−08 [M]
wavelength shift nm
0.0
0.1
0.2
0.3
0.4
0
200
400
600
800
1000
1200
time s
dose nM
500
250
100
50
25
